# Behavioral characterization of early nicotine withdrawal in the mouse: a potential model of acute dependence

**DOI:** 10.1186/s12993-024-00227-0

**Published:** 2024-01-13

**Authors:** Baeksun Kim, Heh-In Im

**Affiliations:** 1 Center for Brain Function, Brain Science Institute (BSI), Korea Institute of Science and Technology (KIST), Hwarang-ro 14-gil 5, Seongbuk-gu, Seoul, 02792 Republic of Korea; 2 Division of Bio-Medical Science & Technology, KIST School, Korea University of Science and Technology (UST), Seoul, 02792 Republic of Korea

**Keywords:** Nicotine, Withdrawal, Acute dependence, Mouse, Behavior, Somatic signs

## Abstract

**Background:**

Clinical and preclinical research have demonstrated that short-term exposure to nicotine during the initial experimentation stage can lead to early manifestation of withdrawal-like signs, indicating the state of “acute dependence”. As drug withdrawal is a major factor driving the progression toward regular drug intake, characterizing and understanding the features of early nicotine withdrawal may be important for the prevention and treatment of drug addiction. In this study, we corroborate the previous studies by showing that withdrawal-like signs can be precipitated after short-term nicotine exposure in mice, providing a potential animal model of acute dependence on nicotine.

**Results:**

To model nicotine exposure from light tobacco use during the initial experimentation stage, mice were treated with 0.5 mg/kg (-)-nicotine ditartrate once daily for 3 days. On the following day, the behavioral tests were conducted after implementing spontaneous or mecamylamine-precipitated withdrawal. In the open field test, precipitated nicotine withdrawal reduced locomotor activity and time spent in the center zone. In the elevated plus maze test, the mecamylamine challenge increased the time spent in the closed arm and reduced the number of entries irrespective of nicotine experience. In the examination of the somatic aspect, precipitated nicotine withdrawal enhanced the number of somatic signs. Finally, nicotine withdrawal did not affect cognitive functioning or social behavior in the passive avoidance, spatial object recognition, or social interaction test.

**Conclusions:**

Collectively, our data demonstrate that early nicotine withdrawal-like signs could be precipitated by the nicotinic antagonist mecamylamine in mice, and that early withdrawal from nicotine primarily causes physical symptoms.

**Supplementary Information:**

The online version contains supplementary material available at 10.1186/s12993-024-00227-0.

## Introduction

Cigarette smoking is the leading cause of preventable death worldwide and thus smoking cessation is a critical step for improving global health [1, 2]. Nicotine is a psychoactive chemical that can be found in tobacco, causing neurobehavioral responses such as arousal, pleasure, mood/cognitive changes, appetite suppression, and physical signs [3]. Nicotine can act on neuronal nicotinic acetylcholine receptors (nAChRs) in the brain, which are ligand-gated cation channels that are activated and desensitized in response to nicotine binding. Although our understanding of nicotine physiology is still limited, through acting on various nAChR subtypes in the mesolimbic reward pathway (i.e. ventral tegmental area to nucleus accumbens) and habenulo-interpeduncular pathway, nicotine is thought to exert its reinforcing and aversive effects, respectively, that ultimately contribute to nicotine addiction and continued cigarette smoking [4, 5].

Interestingly, while the chronic intake of nicotine is required to develop addiction, both clinical and preclinical studies have shown that “acute dependence”-like symptoms from nicotine (i.e., signs of nicotine withdrawal and tolerance) emerge even with a low level of nicotine intake [6–13]. In both DSM-V and DSM-V-TR [14], it has been acknowledged that nicotine withdrawal can occur in adolescent smokers even prior to daily tobacco use, and that significant symptoms of nicotine withdrawal can occur in nondaily smokers. In the clinic [7, 15], people frequently report symptoms of withdrawal after their first cigarette, and most smokers report the experience of withdrawal symptoms even before progressing to daily smoking. These findings collectively indicate that nicotine can induce acute dependence in animals. However, while the existence of acute dependence appears undisputable, the behavioral phenotype and pathophysiological significance of acute dependence are still unclear.

In prior studies, rat models of early nicotine withdrawal have been characterized [8, 10, 12], in which reward function and somatic signs were assessed. In this paper, we strived to model and characterize the physical, affective, and cognitive functions during early withdrawal from nicotine in mice, thereby supplying a novel preclinical model of acute dependence. Mimicking light nicotine intake during the initial experimentation stage of cigarette use in novice smokers [7, 16], low-dose nicotine (0.5 mg/kg (-)-nicotine ditartrate, which is nearly equivalent to 0.175 mg/kg free-base nicotine) was systemically administered to mice once daily for three days. The dosage of nicotine was decided based on previous studies showing that intraperitoneal administration of 0.175 mg/kg nicotine to mice should be sufficient to evoke striatal dopamine release and induce behavioral alterations in wild-type mice [17–19]. It has been proven that abrupt pharmacological reversal of a drug’s action through inactivation of the target receptors in drug-dependent animals leads to the rapid and predictable emergence of withdrawal-like behaviors [20, 21], such as in the case of naloxone for opioid withdrawal [22–24]. In the case of nicotine withdrawal, administration of the nicotinic antagonist mecamylamine allows experimental control over the onset timing, symptom severity, and replicable measurements of nicotine withdrawal in rodents [8, 25]. As such, mice were challenged with either saline or mecamylamine to elicit spontaneous or precipitated signs of nicotine withdrawal, respectively [21, 25, 26].

Important validity criteria in modeling precipitated drug withdrawal are that (1) the signs of withdrawal should be precipitated by antagonist administration in drug-exposed animals and not in drug-naïve animals, and that (2) the withdrawal signs should be higher/larger in animals after precipitated drug withdrawal than in animals after spontaneous drug withdrawal [21, 23]. We explored these two criteria in our mouse model of early nicotine withdrawal using a battery of behavioral assays that encompass physical, affective, and cognitive domains.

## Results

**Fig. 1:**
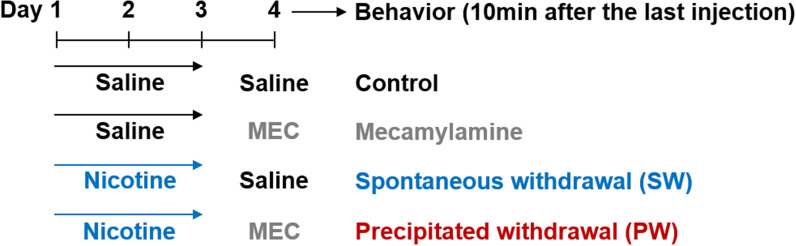
Drug injection and experimentation schedule. Mice were treated with saline or nicotine solution (0.175 mg/kg free-base) once daily for three days. On the following day, mice were treated with saline or mecamylamine solution (MEC; 0.3 mg/kg). All behavioral tests commenced 10 min after the last injection (saline or mecamylamine). Mice treated only with saline were designated as the control group (black). Mice treated with three days of saline followed by mecamylamine were designated the mecamylamine (MEC) group (gray). Mice treated with three days of nicotine followed by saline were designated the early spontaneous withdrawal (SW) group (blue). Mice treated with three days of nicotine followed by mecamylamine were designated the early precipitated withdrawal (PW) group (red)

 To mimic nicotine exposure from light cigarette use during the initial experimentation stage, C57BL/6 N wild-type mice were treated with nicotine (0.5 mg/kg (-)-nicotine ditartrate in physiological saline, pH adjusted to 7.4) once daily for three days. On the following day, mice were treated with 0.3 mg/kg mecamylamine (MEC) to induce precipitated withdrawal (PW) from nicotine, while other mice were treated with saline to induce spontaneous withdrawal (SW). Mecamylamine or saline was administered 24 h after the last nicotine administration based on previous findings that the somatic signs of nicotine withdrawal intensify 24–48 h after cessation of nicotine administration [25, 26]. Behavioral tests were conducted 10 min after the last injection of MEC or saline. For all experiments, different mice were used and the experimenter was blinded to the experimental conditions during analysis. The overall injection scheme and experimental schedule are depicted in Fig. 1.

**Fig. 2:**
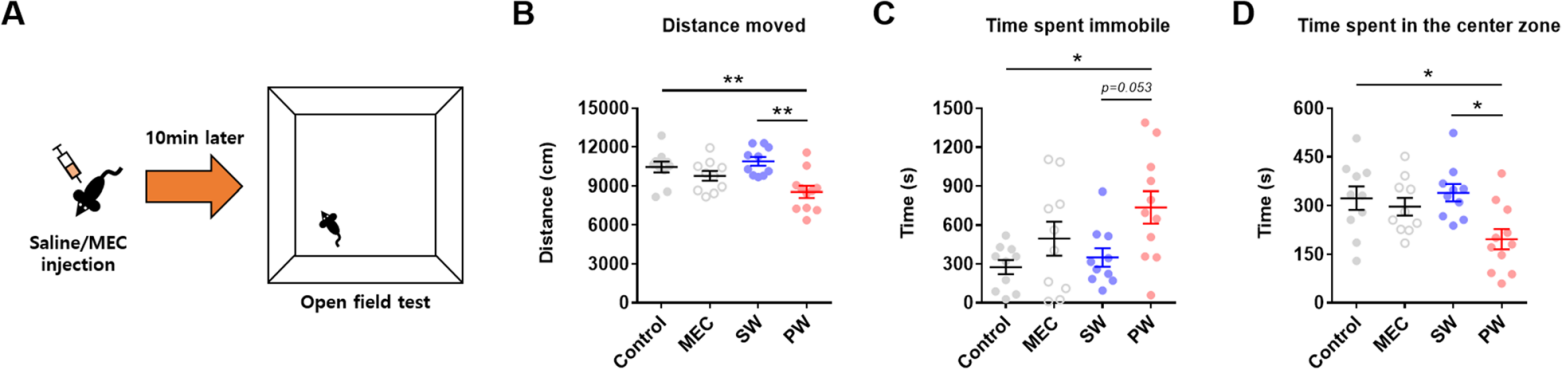
Open field test. **A** Illustration of the open field test. **B** The distance moved was significantly reduced after early precipitated withdrawal (PW) from nicotine (asterisks). **C** The time spent immobile was significantly increased after PW from nicotine (asterisk). **D** The time spent in the center zone was significantly reduced after PW from nicotine (asterisks). Data represent the mean ± S.E.M. from 10–11 mice/group

The open field test was conducted to examine general locomotor function and anxiety-like behavior (Fig. 2A) (*n* = 10–11 mice/group). Precipitated withdrawal from nicotine caused a significant decrease in the distance moved compared to the control and spontaneous withdrawal groups (Fig. 2B) (Group effect, *F*(3,37) = 6.542, *p* = 0.0012; post-hoc analysis, **p = 0.0092 for Control vs. PW, **p = 0.0012 for SW vs. PW). In addition, precipitated nicotine withdrawal led to a significant increase in the time spent immobile compared to the control group (Fig. 2C) (Group effect, *F*(3,37) = 4.024, *p* = 0.0142; post-hoc analysis, *p = 0.0167 for Control vs. PW). Lastly, precipitated nicotine withdrawal significantly reduced the time spent in the center zone compared to the control and spontaneous withdrawal groups (Fig. 2D) (Group effect, *F*(3,37) = 4.600, *p* = 0.0078; post-hoc analysis, *p = 0.0265 for Control vs. PW, *p = 0.0110 for SW vs. PW). These findings show that early precipitated withdrawal from nicotine reduces locomotor activity and increases anxiety-like behavior in the open field, but not early spontaneous withdrawal.

**Fig. 3:**
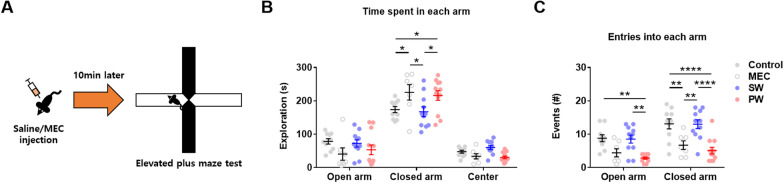
Elevated plus maze test. **A** Illustration of the elevated plus maze test. **B** The time spent in the closed arm was significantly reduced after mecamylamine injection (MEC) or early precipitated withdrawal (PW) from nicotine (asterisks). **C** The number of entries into the open arm was significantly reduced after PW, and the number of entries into the closed arm was significantly reduced after MEC and PW (asterisks). Data represent the mean ± S.E.M. from 7–12 mice/group

Next, the elevated plus maze test was conducted to further examine anxiety-like behavior (Fig. 3A) (*n* = 7–12 mice/group). Unexpectedly, mecamylamine challenge and precipitated nicotine withdrawal caused a significant increase in the time spent in the closed arm (Fig. 3B) (Interaction effect, *F*(6,72) = 3.039, *p* = 0.015; post-hoc analysis, *p = 0.0245 for Control vs. MEC, *p = 0.0296 for Control vs. PW, *p = 0.0106 for MEC vs. SW, *p = 0.0120 for SW vs. PW). In addition, mecamylamine challenge and precipitated nicotine withdrawal caused a significant reduction in the number of entries into the closed arm (Fig. 3C) (Group effect, *F*(3,36) = 14.04, *p* < 0.0001; post-hoc analysis, **p = 0.0034 for Control vs. MEC, ****p < 0.0001 for Control vs. PW, **p = 0.0033 for MEC vs. SW, ****p < 0.0001 for SW vs. PW). On the other hand, only precipitated nicotine withdrawal caused a significant reduction in the number of entries into the open arm (Fig. 3C) (post-hoc analysis, **p = 0.0014 for Control vs. PW, **p = 0.0018 for SW vs. PW). These findings indicate that mecamylamine acutely increases anxiety-like behavior and reduces movement in the elevated plus maze.

Then, the somatic signs of early nicotine withdrawal were assessed to further examine the physical aspects. Previous studies have shown that the somatic signs of nicotine withdrawal in rodents include rearing, head shakes, forelimb shakes (paw tremor), body shakes, jumping, abdominal constrictions, teeth chattering/chewing, facial tremor, scratching, grooming, eye blinks, ptosis, genital licking, yawns, immobility, etc. [21, 25, 26]. Previous clinical studies have demonstrated that the reduction in hand steadiness or increased hand tremor is a prominent motor sign of nicotine withdrawal in humans [27], while macroscopic physical gestures such as head/body shakes and immobility can be readily translated into the clinic. However, most other somatic signs defined in rodents cannot be translated into the physical symptoms of nicotine withdrawal in humans, since those somatic signs are (1) not observed in the clinic, (2) largely rodent-specific, or (3) more appropriate when included in the category of natural rodent behavior. Moreover, preclinical data from pioneering studies have suggested that paw tremor is the single most replicable somatic sign of withdrawal in rodents observed after both low- and high-dose nicotine treatment [21, 25, 26]. Lastly, a seminal study has shown that episodes of locomotor immobility can be observed after precipitated nicotine withdrawal [21]. Therefore, three replicable and translatable signs of somatic nicotine withdrawal were selected for analysis: paw tremors, body shakes, and immobility.

**Fig. 4:**
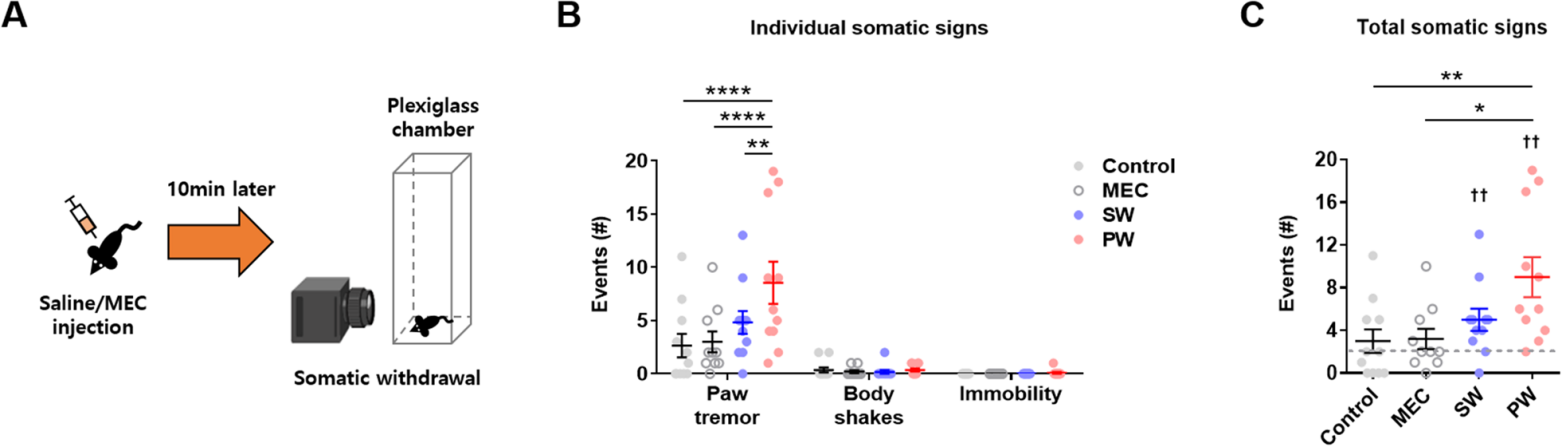
Somatic withdrawal signs. **A** Illustration of the somatic withdrawal sign examination. **B** The number of paw tremors was significantly increased after early precipitated withdrawal (PW) from nicotine (asterisks). **C** The total number of somatic signs was significantly increased after PW and early spontaneous withdrawal (SW) from nicotine (asterisks). The total number of somatic signs was significantly different from the hypothetical value 2 after SW and PW (crosses). Data represent the mean ± S.E.M. from 10–11 mice/group

In the analysis of the somatic signs of early nicotine withdrawal (Fig. 4A) (*n* = 10–11 mice/group), precipitated withdrawal from nicotine caused a significant increase specifically in the number of paw tremors compared to all other groups (Fig. 4B) (Group effect, *F*(3,39) = 4.540, *p* = 0.0080; Interaction effect, *F*(6,78) = 3.643, *p* = 0.0031; post-hoc comparison, ****p < 0.0001 for Control vs. PW, ****p < 0.0001 for MEC vs. PW, **p = 0.0042 for SW vs. PW). In addition, precipitated withdrawal from nicotine caused a significant increase in the overall number of somatic signs compared to the control and mecamylamine challenge groups (Fig. 4C) (Group effect, *F*(3,39) = 4.540; *p* = 0.0080; post-hoc comparison, *p = 0.0134 for Control vs. PW, *p = 0.0185 for MEC vs. PW). Additionally, both spontaneous and precipitated withdrawal from nicotine caused a significant increase in the overall number of somatic signs compared to a hypothetical value of 2 (the value was decided as the median of the control group, which was 2) (Fig. 4C) (SW, sum of signed ranks (W) = 49, ^††^*p* = 0.0098; PW, sum of signed ranks (W) = 55, ^††^*p* = 0.0020). Furthermore, precipitated nicotine withdrawal showed a significant distancing from other groups in the cumulative distribution plot of somatic signs (Additional file 1: Fig. S1A). Lastly, precipitated withdrawal from nicotine caused a largely consistent distribution of somatic events throughout time (Additional file 1: Fig. S1B). These findings show that early precipitated withdrawal from nicotine increases the number of somatic signs, mainly paw tremor.

**Fig. 5:**
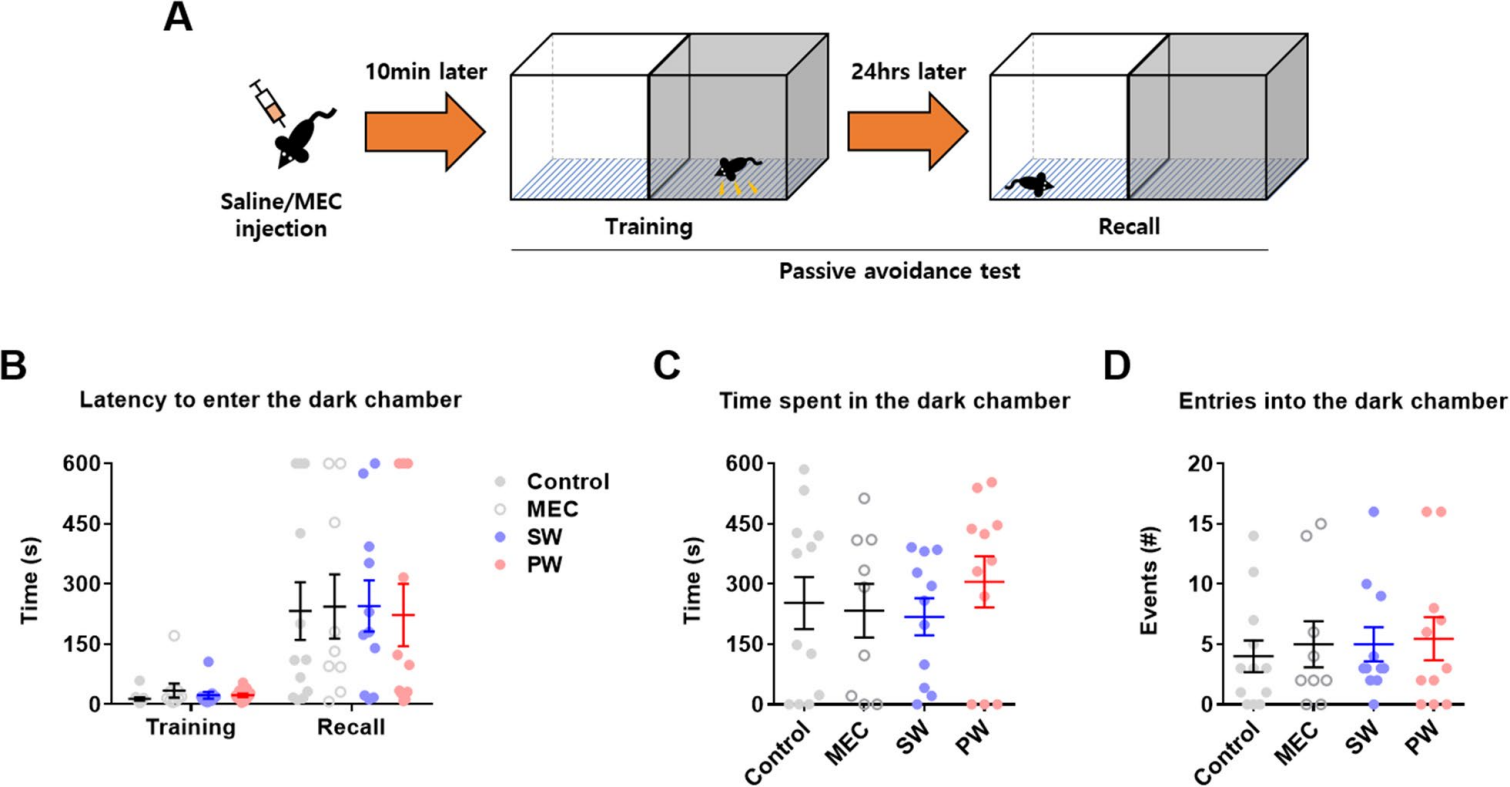
Passive avoidance test. **A** Illustration of the passive avoidance test. **B** The latency to enter the dark chamber did not significantly differ among groups. **C** The time spent in the dark chamber did not significantly differ among groups. **D** The number of entries into the dark chamber did not significantly differ among groups. Data represent the mean ± S.E.M. from 9–12 mice/group

Next, the passive avoidance test was conducted to examine fear memory (Fig. 5A) (*n* = 9–12 mice/group). Early nicotine withdrawal did not alter the latency to enter the dark chamber (Fig. 5B), the time spent in the dark chamber (Fig. 5C), or the number of entries into the dark chamber (Fig. 5D) compared to the other groups. These findings suggest that early withdrawal from nicotine did not affect fear memory.

**Fig. 6:**
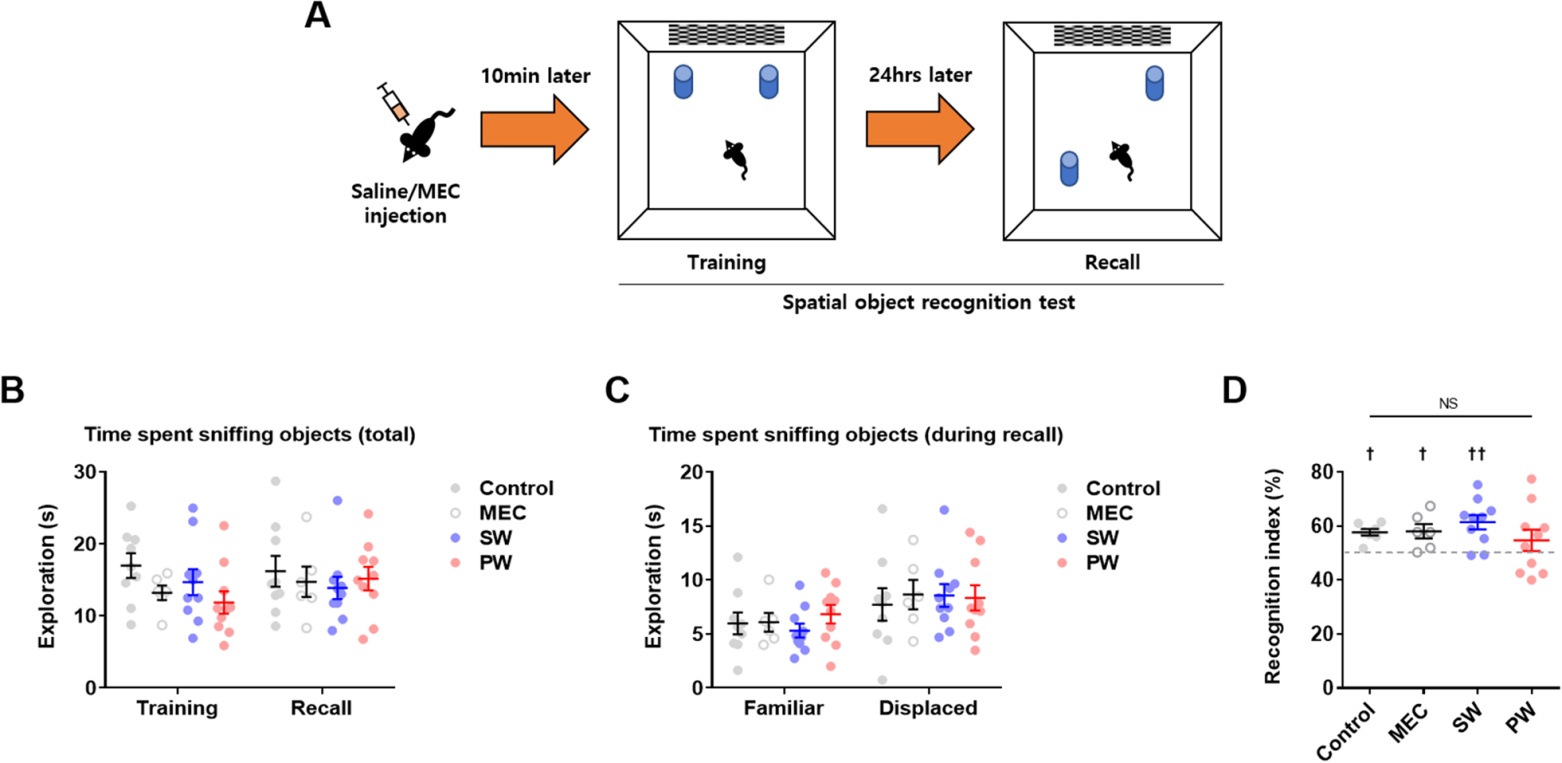
Spatial object recognition test. **A** Illustration of the spatial object recognition test. **B** The total time spent sniffing objects did not significantly differ among groups. **C** The time spent sniffing each object during recall did not significantly differ among groups. **D** The recognition index did not significantly differ among groups (NS: not significant). The recognition index was significantly different from the hypothetical value of 50% in the control group, after mecamylamine injection (MEC), and after early spontaneous withdrawal (SW) from nicotine (crosses). Data represent the mean ± S.E.M. from 6–10 mice/group

Then, the spatial object recognition test was conducted to examine spatial recognition memory (Fig. 6A) (*n* = 6–10 mice/group). Early nicotine withdrawal did not affect the time spent sniffing all objects during either training or recall (Fig. 6B), the time spent sniffing displaced objects during recall (Fig. 6C), or the recognition index (Fig. 6D) compared to other groups. On the other hand, mice after early precipitated withdrawal from nicotine did not differ in the recognition index compared to the hypothetical value of 50% (Fig. 6D) (Control, Sum of signed ranks (W) = 28, ^†^*p* = 0.0156; MEC, Sum of signed ranks (W) = 21, ^†^*p* = 0.0313; SW, Sum of signed ranks (W) = 49, ^††^*p* = 0.0098). These findings suggest that early nicotine withdrawal did not grossly affect spatial recognition memory.

**Fig. 7:**
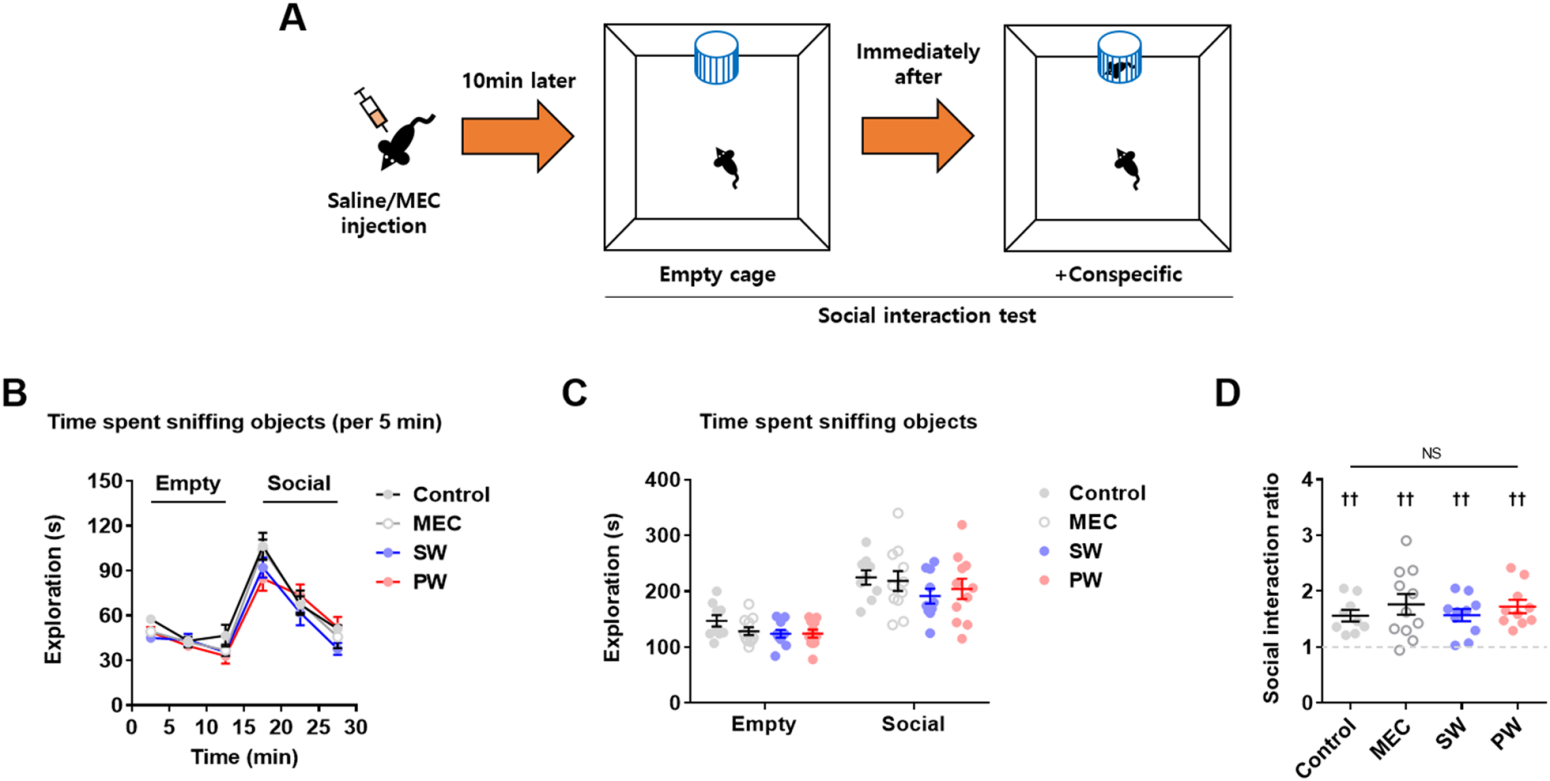
Social interaction test. **A** Illustration of the social interaction test. **B** The time spent sniffing objects did not significantly differ among groups. **C** The total time spent sniffing each object did not significantly differ among groups. **D** The social interaction ratio did not significantly differ among groups (NS: not significant). The social interaction ratio was significantly different from the hypothetical value of 1 in all groups (crosses). Data represent the mean ± S.E.M. from 9–11 mice/group

 Finally, the social interaction test was conducted to examine social behavior (Fig. 7A) (*n* = 9–11 mice/group). Early nicotine withdrawal did not affect the time spent sniffing the empty or social object (Fig. 7B and C), or the social interaction ratio (Fig. 7D) compared to other groups. In addition, early nicotine withdrawal did not affect the social interaction ratio when compared to the hypothetical value of 1 (Fig. 7D) (Control, Sum of signed ranks (W) = 45, ^††^*p* = 0.0039; MEC, Sum of signed ranks (W) = 64, ^††^*p* = 0.0020; SW, Sum of signed ranks (W) = 55, ^††^*p* = 0.0020; PW, Sum of signed ranks (W) = 55, ^††^*p* = 0.0020). These findings suggest that early nicotine withdrawal did not affect social behavior.

## Discussion

This study provides evidence that, in mice, early withdrawal from repeated (3 days), low-dose nicotine (0.175 mg/kg free-base) administration induces physical and affective signs of nicotine withdrawal. Novice smokers do not immediately engage in heavy daily smoking; they usually go through the initial experimentation of smoking through “mooching” or “bumming” [7]. In addition, smokers experience a bolus intake of nicotine, not continuous infusion [26]. This mouse model is significant in that it mimics the initial experimentation stage in human smokers and displays meaningful withdrawal-like signs from short-term nicotine exposure. Although early spontaneous withdrawal from nicotine was not sufficient to induce notable signs of withdrawal (except for somatic signs), a single dose of nicotinic antagonist mecamylamine was able to unmask the latent behavioral signs of early nicotine withdrawal. This suggests that short-term, low-dose nicotine exposure increases dependence vulnerability, or drives animals into an acute dependence-like state.

Mounting evidence suggest that withdrawal signs can be precipitated upon short-term nicotine exposure. A seminal study demonstrated that precipitated withdrawal can ensue even after a single dose of nicotine [8]. In the study, mecamylamine was administered 2 h after a single dose of nicotine in rats. The modeling resulted in a significant elevation of intracranial self-stimulation threshold and somatic signs, which lasted for 5 days after mecamylamine-induced precipitation of nicotine withdrawal. These results showed that acute dependence is a replicable and prominent component of nicotine physiology. Our study further supports the existence of acute dependence to nicotine by showcasing a novel mouse model of early nicotine withdrawal, in which the physical (or somatic) signs were most prominent.

## Addiction versus dependence: the timely question on “acute dependence”

General theories on the transition to addiction dictate that a pattern of chronic, escalating drug intake is required to develop addiction [28, 29]. From an integrative perspective, the hedonic allostasis theory proposes that a spiraling distress cycle takes place during the progression towards drug addiction, in which drug-dependent subjects experience three distinct stages in repetition; preoccupation/anticipation, binge/intoxication, and withdrawal/negative affect [30]. These theories suggest that the term “addiction” refers to a relapsing disease defined by long-term drug taking and seeking.

In comparison to addiction, the term “dependence” should be held separate [31, 32] as recognized in DSM-V-TR (March 2022) [14], for consistency and clarity in the terminologies used in the category of substance use disorders. The term “addiction” mainly refers to the pathological condition of compulsive drug-taking that stems from chronic drug use, whereas the term “dependence” traditionally refers to the normal, physical adaptations that result in tolerance and withdrawal symptoms and can stem from any psychoactive drug/medication that affects the CNS. As such, DSM-V-TR described that (1) dependence does not necessarily indicate the presence of addiction, and that (2) withdrawal can ensue without comorbid use disorder in a wide assortment of drugs including tobacco, alcohol, cannabis, sedatives, stimulants, and opioids. Importantly, the hedonic allostasis theory indicates that withdrawal/negative affect is an essential component in the development of drug addiction. Integrating these ideas, it could be inferred that physical dependence precedes, and is an independent driving factor of, drug addiction.

The important question is the onset time of physical dependence. The overarching evidence from the 20th century to this date have demonstrated that both tolerance- and withdrawal-like behaviors can develop with nondaily, repeated or even a single experience of drug/medication [8, 13, 23, 24, 33–38], which has been termed “acute dependence”. The most noteworthy are the cases of “acute dependence” on opioids, in which repeated/single dose of opioid agonist (i.e. morphine) followed by administration of opioid antagonist (i.e. naloxone) can effectively precipitate the symptoms of opioid withdrawal in both humans and animals [22–24, 36, 39], and has also been acknowledged as a diagnostic criterion for opioid withdrawal throughout DSM-IV to DSM-V-TR [14]. Moreover, pioneering studies have suggested that this early manifestation of tolerance/withdrawal symptoms reflects certain initiating factors that may contribute to the development of the full extent of physical dependence [13, 22, 23], which warrants further attention in the field. However, despite the plethora of evidence, the significance of tolerance/withdrawal signs observed during acute dependence has been far neglected to date.

### Behavioral signs of early nicotine withdrawal

The observed signs of early nicotine withdrawal in this study were mild, which can be expected based on the severity of drug withdrawal being correlated with the dose and duration of drug intake. However, the important findings were that (1) short-term nicotine exposure nevertheless induces acute dependence-like signs and that (2) the magnitude of signs from early nicotine withdrawal are comparable to those reported in previous studies. For example, paw tremors were the most prominent somatic sign after early nicotine withdrawal in mice. The number of paw tremors induced by early precipitated withdrawal from nicotine (mean = 8.545) was comparable to those found in pioneering studies that investigated somatic nicotine withdrawal in rodents (mean = 7–10) [21, 25, 26], in which precipitated withdrawal was induced after chronic nicotine exposure.

In the physical aspect, mice displayed decreased locomotor activity in the open field and an increased number of somatic signs after early precipitated withdrawal from nicotine, at levels that were comparable to those observed in the seminal studies by Isola et al. [26] and Damaj et al. [25]. The effects were attributable to the interaction between nicotine exposure and mecamylamine, suggesting that nicotinic antagonism unmasks (or precipitates) the latent physical symptoms of early nicotine withdrawal. Body shakes and immobility were minor somatic signs in mice, although immobility was prominent during the open field test. This indicates that immobility in the open field may reflect the affective aspect due to the mild anxiogenicity of the open field environment.

Regarding the affective aspect, mice displayed increased anxiety-like behavior in the open field test after early precipitated withdrawal from nicotine, but unexpectedly displayed strong anxiety-like behavior in the elevated plus maze test owing to the mecamylamine challenge. Previous studies have consistently demonstrated that nicotine withdrawal causes anxiety-like behaviors [40–42], but have not reported mecamylamine challenge-induced anxiety-like behavior. The differing phenotypes in the open field and elevated plus maze by mecamylamine challenge might be attributable to the relative anxiogenicity of each environment: The open field is mildly anxiogenic, while the elevated plus maze is more anxiogenic [43]. Systemic mecamylamine at 3.0 mg/kg induced anxiety-like behavior in mice, but only when exposed to a strongly anxiogenic environment (i.e., elevated plus maze). In nicotine-naïve animals, mecamylamine microinjection into the dorsal hippocampus was found to have an anxiogenic effect in the elevated plus maze test [44], but subcutaneous mecamylamine injection at 3.0 mg/kg did not affect the time spent in the open arm in the elevated plus maze [25]. Although the gross lack of literature on mecamylamine’s sole effect on control subjects precludes further insight, these results imply that the route of mecamylamine administration might have differential effects on anxiety-like behaviors. Collectively, caution is necessary in the interpretation of anxiety-like behaviors observed during mecamylamine-precipitated nicotine withdrawal.

Regarding cognitive aspects, mice did not display alterations in passive avoidance or spatial object recognition. Previous studies have shown that withdrawal from chronic nicotine treatment impairs learning and memory [45, 46], a phenotype that is distinct from the absence of cognitive dysfunction during early nicotine withdrawal in this study. In addition, mice did not display altered social behavior in the social interaction test after early nicotine withdrawal. A body of clinical studies has suggested that withdrawal from nicotine seems to impair social functioning [47], but whether it could be replicated in rodents has not been investigated to date. At the least, during early nicotine withdrawal, mice do not display overt deficits in social behavior. The lack of cognitive and social phenotypes in early nicotine withdrawal suggests that acute dependence presents a distinct (or at least a less severe) set of behavioral phenotypes compared to that of chronic dependence.

### Limitations of the study

Three limitations of this study warrant caution in the generalization of the findings. First, although the prevalence of cigarette smoking is nearly four times higher in men [48], the importance of nicotine withdrawal in women cannot be overlooked, as the burden of nicotine withdrawal seems to be as crucial in women as in men [49, 50]. In addition, three translatable and replicable somatic signs were analyzed in this study, but examination of all other somatic-like signs (e.g., teeth chattering/chewing, jumping, scratching, etc.) may yield more information about the impact of early nicotine withdrawal on animals. Lastly, the widely used markers of nicotine withdrawal, i.e. blood nicotine and cotinine levels, were not measured. However, this was because blood nicotine and cotinine are not reliable markers of nicotine withdrawal as stated in DSM-V-TR [14], and because nicotine pharmacokinetics is abnormally higher in mice than in humans [51].

Other experimental limitations of this study warrant further investigation. For instance, only a single dose (0.175 mg/kg free-base nicotine) and single duration (three days of daily exposure) regimen was implemented on a single rodent strain, thus further studies should investigate the impacts of nicotine dosage, exposure duration, and genetic influence on early nicotine withdrawal. In addition, the predictive validity of this mouse model has not been explored (i.e. reversal of withdrawal signs by varenicline or bupropion). The main purposes of this study were to demonstrate the existence of early nicotine withdrawal, and to characterize the phenotype of early nicotine withdrawal. Regardless, the therapeutic effect (and lack thereof) of clinically approved drugs on early nicotine withdrawal and its potential difference with withdrawal from chronic nicotine exposure should be confirmed. Also, the attenuation of early withdrawal symptoms by nicotinic agonists was not examined. This was due to the finding that spontaneous early withdrawal did not induce significant withdrawal signs in mice except for somatic signs, which was expected from the short-term low-dose nicotine administration regimen.

## Conclusion

In summary, our study demonstrated that early nicotine withdrawal produces behavioral alterations in mice, supporting the preclinical findings [6–13] and clinical observations [7, 15] that short-term low-dose nicotine can induce an acute dependence-like state in animals. Although the phenotype of acute dependence on nicotine is clear and its presence might indicate potential vulnerability to the progression toward daily smoking, the pathophysiological significance of acute dependence on nicotine has been neglected. We believe that the phenomenon of early nicotine withdrawal deserves more attention in the field. In the future, (1) the neurobiological mechanisms underlying early nicotine withdrawal could be investigated, (2) the molecular/behavioral differences as well as the progression from acute to chronic dependence on nicotine could be explored in depth, and (3) the potential impact of early nicotine withdrawal on the progression to addiction could be assessed.

## Methods

### Animals

Seven- to eight-weeks-old male C57BL/6 N mice were purchased (Daehan Bio Link, Daejeon, Republic of Korea) 1 week before experimentation. Mice were housed in plastic cages with metal wire grids and were maintained under a 12-h reversed light/dark cycle (lights off at 7:00 AM). Mice had ad libitum access to food and drinking water. Mice were housed in groups of 2 to 4. All animals were randomly assigned to each group.

### Induction of early nicotine withdrawal

(-)-Nicotine ditartrate (Cat. No. 3546; Tocris Bioscience, Abindgon, UK) was dissolved in physiological saline (0.175 mg/kg free-base), and the pH was adjusted to 7.4. Mecamylamine hydrochloride (M9020; Sigma-Aldrich, St. Louis, MO, USA) was dissolved in physiological saline (3.0 mg/kg). Mice were intraperitoneally injected with the nicotine solution (10 ml/kg) once/day for three days and were intraperitoneally injected with the mecamylamine solution on the following day (24 h after the last nicotine injection) to precipitate the behavioral signs of early nicotine withdrawal. The dosing regimen is illustrated in Fig. 1.

### Behavioral tests

Mice were handled for more than 3 days (10 min/day) prior to behavioral tests. All behavioral tests were video-recorded for analysis. Each behavioral test was performed with an independent batch of animals. All behavioral tests commenced at 10 min after the last injection of saline or mecamylamine solution. All experiments were replicated at least once. During analysis, the experimenter was blinded to the groups of mice.

### Open field test

The open field test was conducted to measure general locomotor activity and anxiety-like behavior [52]. A white open field box consisting of (in cm; L x W x H) 40 × 40 × 40 inner dimensions was used for the test. The floor luminosity was maintained at 5 lx. Mice were placed facing one side of the wall within the open field box, and allowed to freely explore the box for 30 min. The distance moved in the open field, the time spent immobile in the open field, and the time spent in the center zone (20 × 20 cm) were analyzed using EthoVision XT 11.5 (Noldus, Wageningen, Netherlands).

### Elevated plus maze test

The elevated plus maze test was conducted to measure anxiety-like behavior [53]. An apparatus consisting of an elevated maze with four arms (two white open arms and two black closed arms), each arm consisting of (in cm; L x W) 60 × 10 inner dimensions was used for the test. The closed arms were surrounded by 18-cm-high walls. The center of the elevated plus maze was maintained at 5 lx. The maze was elevated 50 cm above the ground. Mice were placed facing the wall at the end of the closed arm and allowed to freely explore the maze for 5 min. The time spent in each compartment (open arms, closed arms, and center zone) and the number of entries to each arm type were manually analyzed using a stopwatch. An entry was defined as the mouse having three paws into an arm or the center zone of the maze.

### Somatic signs of nicotine withdrawal

Somatic signs were analyzed to measure physical withdrawal symptoms in mice [25, 26]. A clear plexiglass square column consisting of (in cm; L x W x H) 7 × 7 × 30 inner dimensions with openings at the top and bottom was used for measurement of the somatic signs of nicotine withdrawal in mice. The floor luminosity was maintained at 100 lx. Mice were confined in the plexiglass column for 20 min to allow a close-up video-examination of paw and body movements.

The number of events was counted for each sign: paw tremor (rapidly shaking paw(s) two times while the two paws are supported on the ground or columnar wall or three times while three paws are in support), body shakes (wet-dog shakes; rapidly shaking the body with the anteroposterior axis as the axis of rotation), and freezing (continuous immobility with minimal movement and without paw movement for 60 s). For paw tremors or body shakes, (1) the events that occurred within 10 s of each other were counted as a single event (10-s epoch), and (2) the events that appeared 3 s before or after grooming were excluded from analysis (counted as an innate sequence for grooming).

### Passive avoidance test

The passive avoidance test was conducted to measure fear memory [53]. A two-chambered foot-shock apparatus (Jeungdo Bio & Plant Co., Seoul, Republic of Korea) consisting of light (~ 100 lx) and dark chambers separated by a gate was used for the test. Mice were gently placed in the light chamber, and the gate was opened after 1 min. When mice entered the dark chamber, the gate was closed, and an electrical foot-shock (0.2 mV, 2 s) was delivered through the floor grid. Mice were left in the dark compartment for an additional 1 min and then returned to the home cages. On the following day, mice were placed in the light chamber, the gate was opened after 1 min, and mice were allowed to freely explore the two chambers for 10 min. The latency to enter the dark chamber, the time spent in the dark chamber, and the number of entries into the dark chamber were manually analyzed. An entry was defined as the mouse having all four paws into one chamber.

### Spatial object recognition test

The spatial object recognition test was conducted to measure spatial recognition memory [54]. The open field box, two identical objects (blue glossy cylinder) consisting of 7-cm height and 4-cm radius, and a visual cue consisting of (in cm; L × W) 18 × 24 dimensions with a checkered pattern of (in cm) 2 × 2 dimensions were used for the test. The visual cue was attached to one wall of the open field box.

On the training day, the two objects were placed in the corner, 8 cm away from each wall, near the visual cue-attached wall (Fig. 6A, middle). On the recall day (24 h after training), one of the two objects placed during the training day was moved perpendicular from its original position and to the opposite wall of the visual cue-attached wall (Fig. 6A, right). For both training and recall, mice were placed facing the opposite side of the visual cue-attached wall within the open field box, and allowed to freely explore the box for 10 min. The time spent sniffing each object was manually analyzed using a stopwatch, and the recognition index was calculated. The recognition index, defined in a previous study [55], is as follows:

$$Recognition \,index=\frac{{T}_{d}}{\left({T}_{d}+{T}_{f}\right)}\times 100\%$$

Here, *T*_*d*_ is the time spent exploring the displaced object, and *T*_*f*_ is the time spent exploring the familiar object.

### Social interaction test

The social interaction test was conducted to measure social behavior [56]. The open field box and a cylindrical stainless steel cage measuring 15-cm high and 5-cm wide (radius) were used for the test. The cage was placed near one wall of the open field box in the central position. During the first session, the cage remained empty. During the next session, a conspecific weighing ~ 90% of the exploring mouse’s body weight was confined in the cage. The two sessions were carried out consecutively. For both sessions, mice were placed facing the opposite side of the wall with a stainless steel cage within the open field box and allowed to freely explore the box for 15 min. The time spent sniffing the cage was manually analyzed, and the social interaction ratio was calculated. The social interaction ratio, as in a previous study [56], was defined as follows:

$$Social \,interaction \,ratio=\frac{{T}_{c}}{{T}_{e}}$$

Here, *T*_*c*_ is the time spent exploring the conspecific-containing cage, and *T*_*e*_ is the time spent exploring the empty cage.

### Statistics

One-way analysis of variance (ANOVA) followed by Holm-Sidak’s post-hoc test (Figs. 2, 4C, 5C and D, 6D and 7D) and two-way repeated measures (RM) ANOVA followed by Holm-Sidak’s post-hoc test (Figs. 3, 4B, 5B, 6B and C and 7B and C) were conducted to identify between-subject differences in behavior. The Wilcoxon signed rank test (Figs. 4C, 6D and 7D) was conducted to identify the differences between one sample and a specified hypothetical value. *p* < 0.05 was considered statistically significant. Exact *p* values, *F* values, degrees of freedom, and the sum of signed ranks (W) are provided in the manuscript. Data are displayed as the mean ± standard error of the mean (SEM). Statistical analyses were performed with Prism v6.0 (GraphPad, CA, USA).

### Study approval

All procedures regarding the handling and use of animals in this study were conducted as approved by the Institutional Animal Care and Use Committee (IACUC) of the Korea Institute of Science and Technology (KIST).

### Supplementary Information

**Additional file 1: Figure S1. **Further examination of the somatic withdrawal signs. (A) The cumulative distribution plot of somatic signs after early precipitated withdrawal (PW) from nicotine was notably distanced from those of all other groups. (B) Raster plot of somatic signs.

## Data Availability

All datasets supporting the findings of this study are available within the article. Source data can be provided from the corresponding author upon request.
