# Supplementary figures and images for: Behavioral characterization of early nicotine withdrawal in the mouse: a potential model of acute dependence
Source: Behav Brain Funct. 2024 Jan 13;20:1. doi: 10.1186/s12993-024-00227-0 (PMC10788015; doi:10.1186/s12993-024-00227-0)

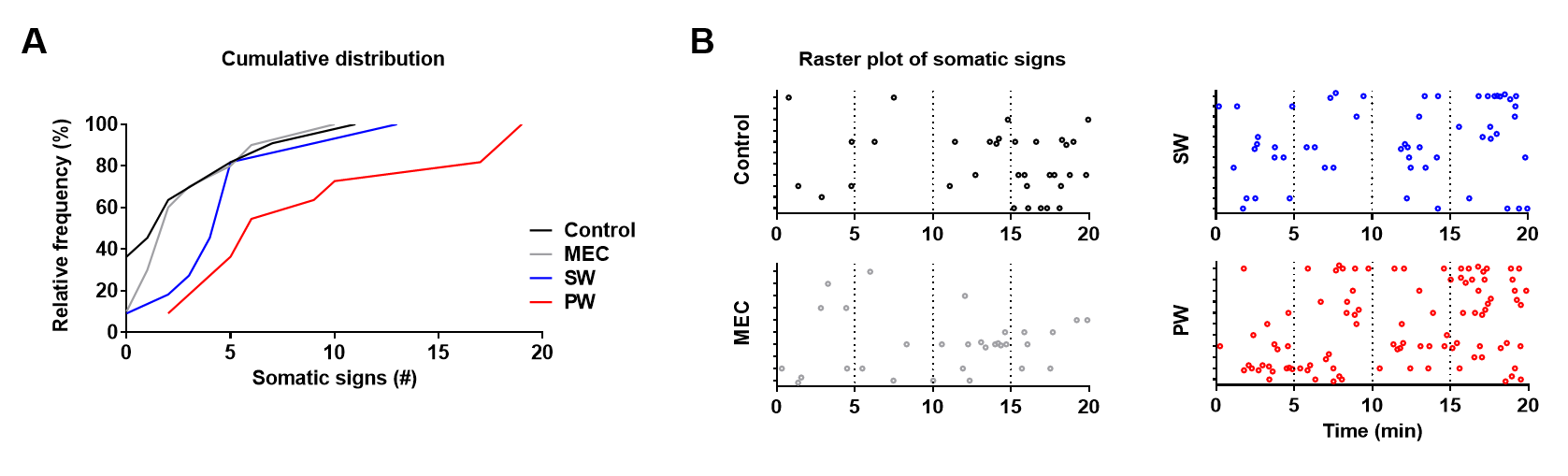

Supplement: Supplementary file 1 — Additional file 1: Figure S1. Further examination of the somatic withdrawal signs. (A) The cumulative distribution plot of somatic signs after early precipitated withdrawal (PW) from nicotine was notably distanced from those of all other groups. (B) Raster plot of somatic signs. [file 12993_2024_227_MOESM1_ESM.tif]
